# Supplementary material for: Measuring glycolytic flux in single yeast cells with an orthogonal synthetic biosensor
Source: Mol Syst Biol. 2019 Dec 19;15(12):e9071. doi: 10.15252/msb.20199071 (PMC6920703; doi:10.15252/msb.20199071)
Supplement: Supplementary file 1 — Appendix [file MSB-15-e9071-s001.docx]

**Appendix**

**Table of Contents**

**Appendix Table S1.** List of the position of the cis-regulatory element sequences.

**Appendix Table S2.** List of the synthesized cis-regulatory elements.

**Appendix Table S3.** Detailed motivations for the introduced mutations.

**Appendix Table S4.** Comparison of predicted mutant behaviors and actually observed ones.

**Appendix Table S5.** List of the plasmids used and generated in this work.

**Appendix Table S6.** Primers list for the construction of the CggR cis-regulatory reporter plasmid.

**Appendix Table S7.** List of primers used to generate the CggR integrative plasmids.

**Appendix Table S8.** List of yeast strains used and generated in this work.

**Appendix Table S9.** Primers used for CggR site-directed mutagenesis.

**Appendix Table S10.** Metabolites that were added to the network model.

**Appendix Table S11.** Additional reaction of the metabolic network model.

**Appendix Table S12.** Intracellular metabolite concentrations of *S. cerevisiae* WT and TM6.

**Appendix Table S13.** Physiological parameters used for the intracellular metabolic flux prediction.

**Appendix Figure S1.** The ratio of CggR bound to DNA at 20 mM FBP *versus* the one at 0 mM FBP represents the FBP-dependent modulation of the CggR-DNA-binding.

**Appendix Figure S2.** mCherry intracellular levels (abundance) linearly correlate with CggR intracellular levels in wildtype (WT) and TM6 strains.

**Appendix Figure S3.** Emission spectra of employed fluorescent proteins and applied filters applied for our flow cytometer indicate only marginal spectral overlap.

**Appendix Figure S4.** Alignment of the four designed and tested promoter variants.

**Appendix Figure S5.** Cellular growth rate is not affected by the expression of the flux-sensor construct in WT (A) and TM6 (B) cells.

**Appendix Figure S6.** The flux-sensor output shows no correlation with the cellular growth rate.

**Appendix Figure S7.** R250A intracellular levels (abundance) linearly correlate with wildtype CggR intracellular levels in WT and TM6 strains.

**Appendix Figure S8.** Unregulated controls of the glycolytic flux sensor.

**Appendix Figure S9.** Results of the regression analysis for the six growth conditions of wildtype and TM6 strain.

**Appendix Figure S10.** The production rates of YFP and mCherry are uncoupled during the cell cycle.

| **Appendix Table S1.** List of the position of the cis-regulatory element sequences.   \| **Element** \| **Position** \| **Sequence** \| \| --- \| --- \| --- \| \| 5’ – end \| 1 -100 \| CTGTCGATTCGATACTAACGCCGCCATCCAGTGTCGAAAACGAGCTCTCGAGAACCCTTAATATAACTTCGTATAATGTATGCTATACGAAGTTATTAGG \| \| CggRO L I \| 135-140  224-229  318-323 \| CGGGAC \| \| CggRO L II \| 147-150  236-239  330-333 \| TGTC \| \| CggRO R I \| 155 -160  244-249  338-343 \| CGGGAC \| \| CggRO R II \| 167-170  256-259  350-353 \| TGTC \| \| TATA box \| 190-197  292-299 \| TATATATA  & TTTATATA \| \| 5’UTR of P_CYC1_*1* \| 368-417 \| ACTATACTTCTATAGACACACAAACACAAATACACACACTAAATTAATAA \| \| 3’-end \| 418-562 \| ATGTCTAAAGGTGAAGAATTATTCACTGGTGTTGTCCCAATTTTGGTTGAATTAGATGGTGATGTTAATGGTCACAAATTTTCTGTCTCCGGTGAAGGTGAAGGTGATGCTACTTACGGTAAATTGACCTTAAAATTTATTTGTA \|   **Appendix Table S2.** List of the synthesized cis-regulatory elements.   \| **Promoter element** \| **Sequence** \| \| --- \| --- \| \| Core-promoter of CYC1 \| TGCGTCAATCGTATGTGAATGCTGGTCGCTATACTGCTGTCGATTCGATACTAACGCCGCCATCCAGTGTCGAAAACGAGCTCTCGAGAACCCTTAATATAACTTCGTATAATGTATGCTATACGAAGTTATTAGGTGATATCAGATCCACTAGTGGCCTATGCTCCTCGAGCAGATCCGCCAGGCGTGTATATATAGCGTGGATGGCCAGGCAACTTTAGTGCTGACACATACAGGCATATATATATGTGTGCGACGACACATGATCATATGGCATGCATGTGCTCTGTATGTATATAAAACTCTTGTTTTCTTCTTTTCTCTAAATATTCTTTCCTTATACATTAGGACCTTTGCAGCATAAATTACTATACTTCTATAGACACACAAACACAAATACACACACTAAATTAATAAATGTCTAAAGGTGAAGAATTATTCACTGGTGTTGTCCCAATTTTGGTTGAATTAGATGGTGATGTTAATGGTCACAAATTTTCTGTCTCCGGTGAAGGTGAAGGTGATGCTACTTACGGTAAATTGACCTTAAAATTTATTTGTA \| \| Core-CYC1 with cggRO \| CTGTCGATTCGATACTAACGCCGCCATCCAGTGTCGAAAACGAGCTCTCGAGAACCCTTAATATAACTTCGTATAATGTATGCTATACGAAGTTATTAGGGAAGACCAAAGCGCCAGTTCATTTGGCGAGCGTTCGGGACTGGATCTGTCCCACCGGGACGCAATCTGTCAGCAGATCCGCCAGGCGTGTATATATAGCGTGGATGGCCAGGCAACTTTAGTGCGGGACCATACATGTCTATACGGGACTGTGCGTGTCCACATGATCATATGGCATGCATGTGCTCTGTATGTATATAAAACTCTTGTTTTCTTCTCGGGACTAAATATGTCTTCGCGGGACATTAGGTGTCTTGCAGCATAAATTACTATACTTCTATAGACACACAAACACAAATACACACACTAAATTAATAAATGTCTAAAGGTGAAGAATTATTCACTGGTGTTGTCCCAATTTTGGTTGAATTAGATGGTGATGTTAATGGTCACAAATTTTCTGTCTCCGGTGAAGGTGAAGGTGATGCTACTTACGGTAAATTGACCTTAAAATTTATTTGTA \| \| syn CggRO promoter V1 \| CTGTCGATTCGATACTAACGCCGCCATCCAGTGTCGAAAACGAGCTCTCGAGAACCCTTAATATAACTTCGTATAATGTATGCTATACGAAGTTATTAGGAACATCGTAACAATACTAAATCAAAAATTGACGCCGGGACCAAGACTGTCTTAACGGGACGTTTACTGTCGATCCTATTGAGTAAATCATATATATAGAGAGCTGGTTTATTAGAATTCCTAACGGGACCTTCGCTGTCAGTCCGGGACTGTAATTGTCCGACATACTCACAAAGTGATAGGCCATTCATTTTTATATAAACGAGGTCAAGTGATCACGGGACTGTCCCTGTCATCGCGGGACTACCCTTGTCATTTGAGAGTTACAACTATACTTCTATAGACACACAAACACAAATACACACACTAAATTAATAAATGTCTAAAGGTGAAGAATTATTCACTGGTGTTGTCCCAATTTTGGTTGAATTAGATGGTGATGTTAATGGTCACAAATTTTCTGTCTCCGGTGAAGGTGAAGGTGATGCTACTTACGGTAAATTGACCTTAAAATTTATTTGTA \| \| syn CggRO promoter V38 \| CTGTCGATTCGATACTAACGCCGCCATCCAGTGTCGAAAACGAGCTCTCGAGAACCCTTAATATAACTTCGTATAATGTATGCTATACGAAGTTATTAGGGACATCGTAACAACACCAAACCAAAACTTGGCGACGGGACCAGGACTGTCTTACCGGGACGTTTACTGTCGCTCCTGTTGAGTGAATCATATATATAGAAAGCGGGTTTTTTTAAATTCCTAACGGGACTTTTTTTGTCAGTCCGGGACTTTAATTGTCCGAAAAAATAAAAAAGTGATAGGGTATATATTTTTATATAAACGAGGTTAAGTGATCGCGGGACTTTCCCTGTCATCGCGGGACTACCCTTGTCATTTGAGATTAAAAACTATACTTCTATAGACACACAAACACAAATACACACACTAAATTAATAAATGTCTAAAGGTGAAGAATTATTCACTGGTGTTGTCCCAATTTTGGTTGAATTAGATGGTGATGTTAATGGTCACAAATTTTCTGTCTCCGGTGAAGGTGAAGGTGATGCTACTTACGGTAAATTGACCTTAAAATTTATTTGTA \|   **Appendix Table S3.** Detailed motivations for the introduced mutations. | |  |
| --- | --- | --- | --- | --- | --- | --- | --- | --- | --- | --- | --- | --- | --- | --- | --- | --- | --- | --- | --- | --- | --- | --- | --- | --- | --- | --- | --- | --- | --- | --- | --- | --- | --- | --- | --- | --- | --- | --- | --- |
| **Mutation** | **Expected effects of the mutations** | |
|  |  |  |
| **T151S** | T151 makes a hydrogen-bond via its hydroxyl hydrogen atom to a terminal oxygen of the 6-phosphate group of FBP. This H-bond appears to be strong, as evidenced by an oxygen-oxygen distance of 2.6 Å in the 3BXF structures. The T151S mutation could preserve this H-bond bond as the hydroxyl group of a serine can be positioned at the same spot as that of a threonine, although less rigidly due to the deletion of the methyl group of the threonine. Thus, this mutation is expected to have **negligible to mild** effects on binding affinity | |
| **T151V** | This mutation introduces an isosteric replacement for the original threonine, and is thus expected to eliminate the above-mentioned strong H-bond and decrease binding affinity for FBP. One would therefore expect to see a **mild to strong** effect on the binding affinity for FBP. | |
| **T152S** | Like T151, the hydroxyl side chain of T152 makes a relatively strong H-bond to one of the terminal oxygens of the 6-phosphate of FBP (oxygen-oxygen distance 2.6 Å). With the same rationale as for T151S, the effect of the mutation of FBP binding is expected to be **negligible to mild**. | |
| **R175K** | The positively charged R175 side chain makes a strong (nitrogen-oxygen distance 2.7 Å) H-bond with a terminal oxygen of the 1-phosphate group of FBP. Mutating the arginine to a lysine may give less strong interactions to the FBP, resulting in decreased affinity. A complicating factor is that R175 radically changes side chain orientation upon binding of FBP due to a G177-Q185 loop movement in the protein. The FoldX predictions suggest that for the R175K mutant the equilibrium may shift to the FBP conformation also in the absence of FBP. Thus, the effect of the mutation could vary from **negligible to strong.** | |
| **R250A** | R250 makes two H-bonds with the 1-phosphate group of FBP. The N-O distances are 3.3 and 2.9 Å. One H-bond is to a terminal phosphate oxygen, the other is to the ether oxygen (in between the phosphor and the carbon atom). One would expect the overall effect on binding to be strong unless the created cavity allows for water molecules to come in and replace the hydrogen bonds that were lost by removing the R250 side chain. Thus, mutation of this residue could give rise to a **mild to strong** affinity decrease. | |
| **E269Q** | The side chain of E269 accepts H-bonds for the hydroxyl groups at the 3 and 4 positions of the FBP. The oxygen-oxygen distances are 2.6 and 2.5 Å, which indicates strong H-bonding interactions. Mutation to glutamine would possibly only change the H-bond pattern but not decrease the overall number of H-bonds. This would then still be expected to weaken the interactions as the newly formed H-bonds would be less polarized than those in the wild-type structure. Thus, the expected result of this mutation is a **mild to strong** affinity decrease for FBP | |

**Appendix Table S4.** Comparison of predicted mutant behaviors and actually observed ones.

| **Mutation** | **Expected effects on affinity for FBP** (and if relevant on equilibrium and stability)^a^ | **Obtained effects on affinity for FBP** | **FoldX predicted stability changes** (kJ/mol)^b^ | | | **Obtained effects on stability** | **Obtained effects on DNA binding affinity** |  |
| --- | --- | --- | --- | --- | --- | --- | --- | --- |
|  |  |  | ∆∆G^fold^ for the normal-conformation | ∆∆G^fold^ for the activated conformation | ∆∆∆G^fold^ (between the conformations) |  |  |  |
| **T151S** | **negligible to mild** affinity decrease | **mild** affinity  increase | -1.4 | -1.3 | 0.1 | No effect | No effect | |
| **T151V** | **mild to strong** affinity decrease | **strong** affinity decrease | 2.5 | 3.1 | 0.5 | **Strong** decrease | **Reduced** affinity | |
| **T152S** | **negligible to mild** affinity decrease | **negligible** affinity increase | 7.3 | 4.6 | -2.7 | No effect | No effect | |
| **R175K** | **negligible to strong** affinity decrease and possibly a **shift of equilibrium** to the activated conformation | **negligible** affinity increase | 16.5^C^ | -0.7 | -17.2^C^ | No effect | **Disrupted** affinity | |
| **R250A** | **mild to strong** affinity decrease | **strong** affinity decrease | 2.2 | 2.7 | 0.5 | No effect | No effect | |
| **E269Q** | **mild to strong** affinity decrease for FBP in combination with **overall destabilization** | **mild** affinity  increase | 16.4^C^ | 11.4 ^C^ | -5.0 | **Strong** decrease | **Disrupted** affinity | |
| ^a^A detailed justification for the expected effects of the mutations on binding affinity is given in Appendix Table S2. ^b^A downshift in ∆∆G^fold^ predicts stabilization of the protein, while a downshift in ∆∆∆G^fold^ predicts that the FBP-conformation becomes more favorable. ∆∆∆G^fold^ represents the difference between the ∆∆G^fold^ values for the two conformations. ^C^Values are significantly higher than the standard deviation of FoldX predictions, which equals 3.4 kJ/mol (Guerois *et al*, 2002). The classification from mild to strong affinity decrease was based on the FBP-CggR binding affinity assessed by thermal shift assays. | | | | | | | | |

**Appendix Table S5.** List of the plasmids used and generated in this work.

| **Plasmid** | **Function** | **Source** |
| --- | --- | --- |
| pET100-CggR-Sc | pET100/D-TOPO plasmid used for CggR protein expression | This study |
| HO-poly-KanMX4-HO | Integrative plasmid | (Voth, 2001) |
| pUG66 | *E. coli* / *S. cerevisiae* shuttle vector containing, Amp+, loxP-bleR-loxP disruption cassette | (Gueldener, 2002) |
| pCM149 | Plasmid for integrative yeast transformation, marker LEU2, CMVp(tetR) | (Bellí *et al*, 1998) |
| p416-loxP-KmR-TEFmut2-yECitrine | CEN/ARS plasmid, loxP-KanMX4-loxP, TEFmut2, yECitrine; Amp+ | (Nevoigt *et al*, 2006) |
| p416-loxP-KmR-TEFmut7-yECitrine | CEN/ARS plasmid, loxP-KanMX4-loxP, TEFmut7, yECitrine; Amp+ | (Nevoigt *et al*, 2006) |
| p416-loxP-KmR-TEFmut6-yECitrine | CEN/ARS plasmid, loxP-KanMX4-loxP, TEFmut6, yECitrine; Amp+ | (Nevoigt *et al*, 2006) |
| p416-loxP-KmR-TEFmut8-yECitrine | CEN/ARS plasmid, loxP-KanMX4-loxP, TEFmut8, yECitrine; Amp+ | (Nevoigt *et al*, 2006) |
| pHO_pCMV_CggR_ble | Plasmid for genomic integration of CggR and P_CMV_ promoter | This study |
| pHO_pTEFmut2_CggR_ble | Plasmid for genomic integration of CggR and P_TEFmut2_ promoter | This study |
| pHO_pTEFmut7_CggR_ble | Plasmid for genomic integration of CggR and P_TEFmut7_ promoter | This study |
| pHO_pTEFmut2_CggR_R250A_ble | Plasmid for genomic integration of CggR and P_TEFmut2_ promoter | This study |
| pHO_pTEFmut7_CggR_R250A_ble | Plasmid for genomic integration of CggR and P_TEFmut7_ promoter | This study |
| pBS35 | Plasmid containing the mCherry ORF sequence | **(Hailey *et al*, 2002)** |
| pWHE601 | Plasmid containing the ADH1 terminator sequence | (Suess *et al*, 2003) |
| pYCplac33 | Yeast centromeric plasmid for fluorescence background correction | (Gietz & Akio, 1988) |
| pTEF6-7 | Constitutively regulated eCitrine and mCherry reporter plasmid | This study |
| pCggRO-reporter | Reporter plasmid with constitutively expressed mCherry and CggR-regulated YFP (eCitrine) by promoter element synCggRO-V38 | This study |
| pCggRO-V1-reporter | Reporter plasmid with constitutively expressed mCherry and CggR-regulated YFP (eCitrine) by promoter element synCggRO-V1 | This study |
| pCggRO-Core-CYC1-reporter | Reporter plasmid with constitutively expressed mCherry and CggR-regulated YFP (eCitrine) by promoter element Core_CYC1 with cggRO | This study |
| pCore-CYC1-reporter | Reporter plasmid with constitutively expressed mCherry and CggR-regulated YFP (eCitrine) by wildtype CYC1 core-promoter | This study |

**Appendix Table S6.** Primers list for the construction of the CggR cis-regulatory reporter plasmid.

| **Primer** | **Sequence** | **Function** |
| --- | --- | --- |
| mCherry-F | AAGTTTTCTAGAAAAATGGTGAGCAAGGGCG | Amplification of mCherry ORF |
| mCherry-R3 | TAGAAGTGTCAACAACGCTACTTGTACAGCTCGTCCATG | Amplification of mCherry ORF |
| mCherry_KpnI_rv | ATTAGGTACCCGAGCTCTCGAGAACCCTTAATATAACTT | Amplification of P_TEFmut8_;  Overlap of the mCherry ORF. P_TEFmut8_ and *ADH1* terminator |
| p416-M5_8-RV2 | CGCCCTTGCTCACCATTTTTCTAGAAAACTTAGATTAGATTGCTATGC | Amplification of P_TEFmut8_ |
| ADH-F | CATGGACGAGCTGTACAAGTAGCGTTGTTGACACTTCTAAATAAGCG | Amplification of *ADH1* terminator |
| mCherry_KpnI_fw | ATATGGTACCAGCGTACGAGCGACCTCATG | Amplification of *ADH1* terminator;  Overlap of the mCherry ORF. P_TEFmut8_ and *ADH1* terminator |
| mCherry_ch_fw | GCTGACCATTATCAACAAAATACTCCAATTGG | Check insertion of the mCherry expression cassette |
| mCherry_ch_rv | CGTAAAGCACTAAATCGGAACCCTAAAGG | Check insertion of the mCherry expression cassette |
| TEF6-7_bb_fw | ATGTCTAAAGGTGAAGAATTATTCACTGGTGTTGTC | Gibson assembly of pCggRO_reporter |
| TEF6-7_bb_rv | TCGACACTGGATGGCGGCGTTAGTATCGAATCGACAG | Gibson assembly of pCggRO_reporter |
| URA3_fw | GTGTGCATTCGTAATGTCTGCCCATTCTGC | Gibson assembly of pCggRO_reporter |
| URA3_rv | ACCAAGGAATTACTGGAGTTAGTTGAAGCA | Gibson assembly of pCggRO_reporter |
| pBR322_fw | TACCAAATACTGTCCTTCTAGTGTAGC | Gibson assembly of pCggRO_reporter |
| pBR322_rv | ACCCGACAGGACTATAAAGATACCA | Gibson assembly of pCggRO_reporter |
| CggR_ch_rv | GGTTGGCCATGGAACTGGC | Sequencing of pCggRO_reporter plasmid |

**Appendix Table S7.** List of primers used to generate the CggR integrative plasmids.

| **Primer** | **Sequence** | **Function** |
| --- | --- | --- |
| HO_fw | ATCCACTAGTGGCCTATGCGCGCCGCCATCCAGTGT | Gibson assembly of pHO_pCMV_CggR_ble |
| HO_rv | TGGGCCAAGCTCCTCGACCTAGCGTACGACGCCATTTTAAGTC | Gibson assembly of pHO_pCMV_CggR_ble |
| pUG66_fw | CCTTACGCATCTGTGCGGTACGCCAGCTGAAGCTTCGT | Gibson assembly of pHO_pCMV_CggR_ble |
| pUG66_rv | TTCGACACTGGATGGCGGCGCGCATAGGCCACTAGTGGA | Gibson assembly of pHO_pCMV_CggR_ble |
| TEF_mut2__CggR_fw | GCGTCGTACGCTAGGTCGAGACGGCTCTAAAGTGCTTC | Gibson assembly of pHO_pTEFmut2_CggR_ble |
| TEF_mut2__CggR_rv | TGATGATGAGAACCCCGCATTTTTCTAGAAAACTTGGATTAGATTG | Gibson assembly of pHO_pTEFmut2_CggR_ble |
| HO_TEF_mut2__fw | AATCCAAGTTTTCTAGAAAAATGCGGGGTTCTCATCAT | Gibson assembly of pHO_pTEFmut2_CggR_ble |
| HO_TEF_mut2__rv | CCGAAGCACTTTAGAGCCGTCTCGACCTAGCGTACGAC | Gibson assembly of pHO_pTEFmut2_CggR_ble |
| TEF_mut7__CggR_fw | GCGTCGTACGCTAGGTCGAGATAGCTTCAAAATGTCTCTACTCC | Gibson assembly of pHO_pTEFmut7_CggR_ble |
| TEF_mut7__CggR_rv | TGATGATGAGAACCCCGCATTTTTCTAGAAAACTTAGATTAGATTGC | Gibson assembly of pHO_pTEFmut7_CggR_ble |
| HO_TEF_mut7__fw | TAATCTAAGTTTTCTAGAAAAATGCGGGGTTCTCATCAT | Gibson assembly of pHO_pTEFmut7_CggR_ble |
| HO_TEF_mut7__rv | TAGAGACATTTTGAAGCTATCTCGACCTAGCGTACGAC | Gibson assembly of pHO_pTEFmut7_CggR_ble |
| CggR_in_CggR_fw | CTCCGCGGCCCGAATTCATATGCGGGGTTCTCATCATCATC | CggR expression cassette |
| CggR_in_CggR_rv | AGCTCGGTACCTCGAGCTATCATTCATCTCTCAACAACTT | CggR expression cassette |
| pCggR_promoter_fw | TTAAAATGGCGTCGTACGCTAGGTCGAGGAGCTTGGC | CggR expression cassette |
| pCggR_promoter_rv | GATGATGATGAGAACCCCGCATATGAATTCGGGCCGCGGAG | CggR expression cassette |
| pCggR_terminator_fw | AAGTTGTTGAGAGATGAATGATAGCTCGAGGTACCGAGCT | CggR expression cassette |
| pCggR_terminator_rv | GTACGAAGCTTCAGCTGGCGTACCGCACAGATGCGTAAGGAGAAAATACC | CggR expression cassette |

**Appendix Table S8.** List of yeast strains used and generated in this work.

| **Strain name** | **Genetic alteration** | **Genomic insertion** | **Plasmid name** | **Function** | **Source** | |
| --- | --- | --- | --- | --- | --- | --- |
| WT_pCggRO | Plasmid | *N.A.* | pCggRO | Wildtype strain containing the pCggRO reporter plasmid for control of the cis-regulatory dependent YFP/mCherry expression | This work | |
| WT_YCplac33 | Plasmid | *N.A.* | pYCplac33 | Wildtype strain containing the YCplac33 plasmid for fluorescence background correction | This work | |
| WT_P_CMV__CggR_pCggRO | Genomic; Plasmid | P_CMV_ and CggR | pCggRO | Wildtype strain containing the CggR under the control of P_CMV_ integrated in the genome and the pCggRO reporter plasmid for biosensor analysis | This work | |
| WT_P_CMV__CggR_pYCplac33 | Genomic; Plasmid | P_CMV_ and CggR | pYCplac33 | Wildtype strain containing the CggR under the control of P_CMV_ integrated in the genome and containing the YCplac33 plasmid for fluorescence background correction | This work | |
| WT_P_TEFmut2__CggR_pCggRO | Genomic; Plasmid | P_TEFmut2_ and CggR | pCggRO | Wildtype strain containing the CggR under the control of P_TEFmut2_ integrated in the genome and the pCggRO reporter plasmid for biosensor analysis | This work | |
| WT_P_TEFmut2__CggR_pYCplac33 | Genomic; Plasmid | P_TEFmut2_ and CggR | pYCplac33 | Wildtype strain containing the CggR under the control of P_TEFmut2_ integrated in the genome and containing the YCplac33 plasmid for fluorescence background correction | This work | |
| WT_P_TEFmut7__CggR_pCggRO | Genomic; Plasmid | P_TEFmut7_ and CggR | pCggRO | Wildtype strain containing the CggR under the control of P_TEFmut7_ integrated in the genome and the pCggRO reporter plasmid for biosensor analysis | This work | |
| WT_P_TEFmut7__CggR_pCggRO-V1 | Genomic; Plasmid | P_TEFmut7_ and R250ACggR | pCggRO-V1 | Wildtype strain containing the CggR under the control of P_TEFmut7_ integrated in the genome and the pCggRO-V1 reporter plasmid for biosensor analysis | This work |  |
| WT_P_TEFmut7__CggR_pCggRO-CoreCYC1 | Genomic; Plasmid | P_TEFmut7_ and R250ACggR | pCggRO-Core-CYC1 | Wildtype strain containing the CggR under the control of P_TEFmut7_ integrated in the genome and the pCggRO-CoreCYC1 reporter plasmid for biosensor analysis | This work |  |
| WT_P_TEFmut7__CggR_pCore-CYC1 | Genomic; Plasmid | P_TEFmut7_ and R250ACggR | pCore-CYC1 | Wildtype strain containing the CggR under the control of P_TEFmut7_ integrated in the genome and the pCore-CYC1 reporter plasmid for biosensor analysis | This work |  |
| WT_P_TEFmut7__CggR_pYCplac33 | Genomic; Plasmid | P_TEFmut7_ and CggR | pYCplac33 | Wildtype strain containing the CggR under the control of P_TEFmut7_ integrated in the genome and containing the YCplac33 plasmid for fluorescence background correction | This work | |
| WT_P_TEFmut2__R250ACggR_pCggRO | Genomic; Plasmid | P_TEFmut2_ and R250ACggR | pCggRO | Wildtype strain containing the mutant R250ACggR under the control of P_TEFmut2_ integrated in the genome and the pCggRO reporter plasmid for biosensor analysis | This work | |
| WT_P_TEFmut2__R250ACggR_pYCplac33 | Genomic; Plasmid | P_TEFmut2_ and R250ACggR | pYCplac33 | Wildtype strain containing the mutant R250ACggR under the control of P_TEFmut2_ integrated in the genome and containing the YCplac33 plasmid for fluorescence background correction | This work | |
| WT_P_TEFmut7__R250ACggR_pCggRO | Genomic; Plasmid | P_TEFmut7_ and R250ACggR insertion | pCggRO | Wildtype strain containing the mutant R250ACggR under the control of P_TEFmut7_ integrated in the genome and the pCggRO reporter plasmid for biosensor analysis | This work | |
| WT_P_TEFmut7__R250ACggR_pYCplac33 | Genomic; Plasmid | P_TEFmut7_ and R250ACggR | pYCplac33 | Wildtype strain containing the mutant R250ACggR under the control of P_TEFmut7_ integrated in the genome and containing the YCplac33 plasmid for fluorescence background correction | This work | |
| TM6_pCggRO | Plasmid | *N.A.* | pCggRO | TM6 strain containing the pCggRO reporter plasmid for control of the cis-regulatory dependent YFP/mCherry expression | This work | |
| TM6_YCplac33 | Plasmid | *N.A.* | pYCplac33 | TM6 strain containing the YCplac33 plasmid for fluorescence background correction | This work | |
| TM6_P_CMV__CggR_pCggRO | Genomic; Plasmid | P_CMV_ and CggR | pCggRO | TM6 strain containing the CggR under the control of P_CMV_ integrated in the genome and the pCggRO reporter plasmid for biosensor analysis | This work | |
| TM6_P_CMV__CggR_pYCplac33 | Genomic; Plasmid | P_CMV_ and CggR | pYCplac33 | TM6 strain containing the CggR under the control of P_CMV_ integrated in the genome and containing the YCplac33 plasmid for fluorescence background correction | This work | |
| TM6_P_TEFmut2__CggR_pCggRO | Genomic; Plasmid | P_TEFmut2_ and CggR | pCggRO | TM6 strain containing the CggR under the control of P_TEFmut2_ integrated in the genome and the pCggRO reporter plasmid for biosensor analysis | This work | |
| TM6_P_TEFmut2__CggR_pYCplac33 | Genomic; Plasmid | P_TEFmut2_ and CggR | pYCplac33 | TM6 strain containing the CggR under the control of P_TEFmut7_ integrated in the genome and containing the YCplac33 plasmid for fluorescence background correction | This work | |
| TM6_P_TEFmut7__CggR_pCggRO | Genomic; Plasmid | P_TEFmut7_ and CggR | pCggRO | Wildtype strain containing the CggR under the control of P_TEFmut7_ integrated in the genome and the pCggRO reporter plasmid for biosensor analysis | This work | |
| TM6_P_TEFmut7__CggR_pYCplac33 | Genomic; Plasmid | P_TEFmut7_ and CggR | pYCplac33 | TM6 strain containing the CggR under the control of P_TEFmut7_ integrated in the genome and containing the YCplac33 plasmid for fluorescence background correction | This work | |
| TM6_P_TEFmut2__R250ACggR_pCggRO | Genomic; Plasmid | P_TEFmut2_ and R250ACggR | pCggRO | TM6 strain containing the mutant R250ACggR under the control of P_TEFmut2_ integrated in the genome and the pCggRO reporter plasmid for biosensor analysis | This work | |
| TM6_P_TEFmut2__R250ACggR_pYCplac33 | Genomic; Plasmid | P_TEFmut2_ and R250ACggR | pYCplac33 | TM6 strain containing the mutant R250ACggR under the control of P_TEFmut2_ integrated in the genome and containing the YCplac33 plasmid for fluorescence background correction | This work | |
| TM6_P_TEFmut7__R250ACggR_pCggRO | Genomic; Plasmid | P_TEFmut7_ and R250ACggR | pCggRO | TM6 strain containing the mutant R250ACggR under the control of P_TEFmut7_ integrated in the genome and the pCggRO reporter plasmid for biosensor analysis | This work | |
| TM6_P_TEFmut7__R250ACggR_pYCplac33 | Genomic; Plasmid | P_TEFmut7_ and R250ACggR | pYCplac33 | TM6 strain containing the mutant R250ACggR under the control of P_TEFmut7_ integrated in the genome and containing the YCplac33 plasmid for fluorescence background correction | This work | |

**Appendix Table S9.** Primers used for CggR site-directed mutagenesis.

| **Mutant** | **Amino acid position** | **Original**  **amino acid** | **Mutated**  **amino acid** | **Primer**  **direction** | **Primer**  **Sequence^a^** |
| --- | --- | --- | --- | --- | --- |
| **T151S** | 151 | Thr | Ser | Forward | 5’- GTT ACT GGT GGT TCT ACT ATT GAA G-3’ |
|  |  |  |  | Reverse | 3’-CAA TGA CCA CCA AGA TGA TAA CTT C-5’ |
| **T151V** | 151 | Thr | Val | Forward | 5’-GTT ACT GGT GGT GTT ACT ATT GAA G-3’ |
|  |  |  |  | Reverse | 3’-CAA TGA CCA CCA CAA TGA TAA CTT C- 5’ |
| **T152S** | 152 | Thr | Ser | Forward | 5’-GCT GTT ACT GGT GGT ACT AGC ATT GAA GCT-3’ |
|  |  |  |  | Reverse | 3’-CGA CAA TGA CCA CCA TGA TCG TAA CTT CGA-5’ |
| **R175K** | 175 | Arg | Lys | Forward | 5’-TTT GTT CCA GCT AAG GGT GGT TT-3’ |
|  |  |  |  | Reverse | 3’-AAA CAA CCT CGA TTC CCA CCA CC-5’ |
| **R250A** | 250 | Arg | Ala | Forward | 5’-AAA ACT ATG GCT CAA GCA AGA AAC ACC CCA-3’ |
|  |  |  |  | Reverse | 3’-TTT TGA TAC CGA GTT CGT TCT TTG TGG GGT-5’ |
| **E269Q** | 269 | Glu | Gln | Forward | 5’-AAC GAT GCT GTT ACC CAG GCT TTC GGT TAC-3’ |
|  |  |  |  | Reverse | 3’-TTG CTA CGA CAA TGG GTC CGA AAG CCA ATG-5’ |

^a^ The mutated amino acid sequence is highlighted in red.

**Appendix Table S10.** Metabolites that were added to an existing thermodynamic-metabolic network model (Niebel *et al*, 2019), which we used here to estimate the intracellular metabolic fluxes.

| **Metabolite** | | **Abreviation** |
| --- | --- | --- |
|  | D-galactose | gal |
|  |  |  |
|  | D-galactose 1-phosphate | gal1p |
|  |  |  |
|  | UDP-galactose | udpgal |
|  |  |  |
|  | Maltose | malt |
|  |  |  |

**Appendix Table S11.** Additional reaction of the metabolic network model. Reactions that were added to an existing thermodynamic-metabolic network model(Niebel *et al*, 2019), which we used here to estimate the intracellular metabolic fluxes.

| **Reaction name** | **Reaction** | **Stoichiometric equation** |
| --- | --- | --- |
| D-Galactose exchange | gal-D_EX | gal[e] <=> |
| Maltose exchange | malt_EX | malt[e] <=> |
| Galactokinase | Galkin | gal[c] + atp[c] <=> adp[c] + gal1p[c] |
| Galactose-1-phosphate uridyl transferase | Gal7 | udpg[c] + gal1p[c] <=> g1p[c] + udpgal[c] |
| UDP-glucose-4-epimerase | Gal10 | udpgal[c] <=> udpg[c] |
| Maltase | MALTGD | h2o[c] + malt[c] <=> (2) glc-D[c] |

**Appendix Table S12.** Intracellular metabolite concentrations of *S. cerevisiae* WT and TM6. The mean (in mM) and standard deviation were calculated from three biological replicates, where each of the biological replicates were sampled three times. For the pyruvate condition, the mean values for WT and TM6 were determined from the values obtained for each strain, but the standard deviations were calculated from the combined samples of WT and TM6.

**Appendix Table S13.** Physiological parameters used for the intracellular metabolic flux prediction.

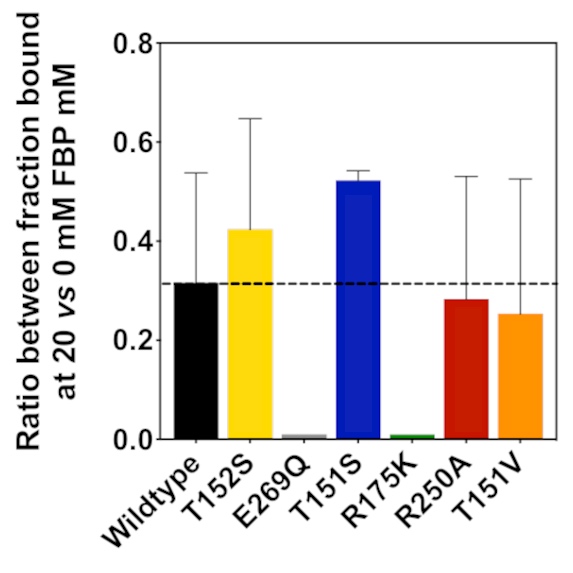


**Appendix Figure S1.** The ratio of CggR bound to DNA at 20 mM FBP *versus* the one at 0 mM FBP represents the FBP-dependent modulation of the CggR-DNA-binding. Error bars correspond to the error calculated from the ratio of the standard deviation of at least three replicates of 0 and 20mM FBP.

**
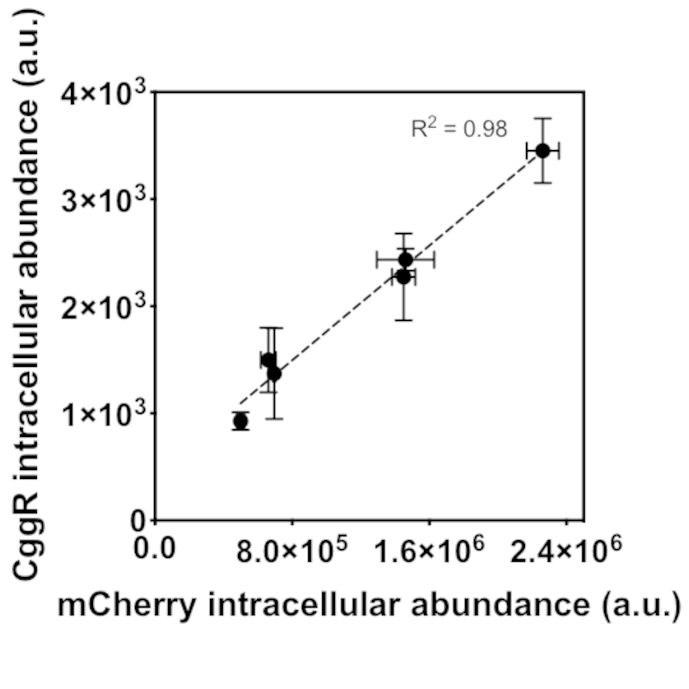
**

**Appendix Figure S2.** mCherry intracellular levels (abundance) linearly correlate with CggR intracellular levels in wildtype (WT) and TM6 strains. mCherry expression was driven by the P_TEFmut8_ and CggR expression was driven by the P_TEFmut7_ promoter. Error bars indicate the standard deviation of three independent replicates.


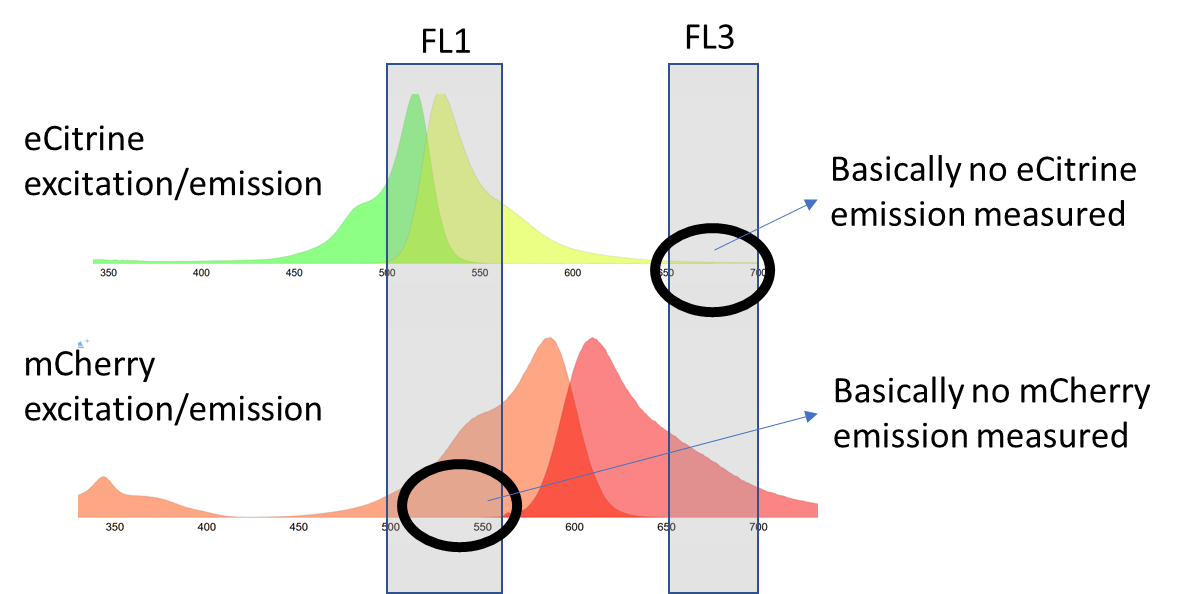


**Appendix Figure S3.** Emission spectra of employed fluorescent proteins and applied filters applied for our flow cytometer indicate only marginal spectral overlap. Excitation and emission spectra were taken from https://www.fpbase.org (Lambert, 2019).

**Appendix Figure S4.** Alignment of the four designed and tested promoter variants. The sequences of the conserved functional elements of the *CYC1* corepromoter and the introduced cggR binding sites are underlined. DNA sequences are highlighted in black and grey for a 100% and 75% match, respectively. The nucleotides of syn CggRO promoter V38 marked in red specify the sequence changes compared to syn CggRO promoter V1, which were introduced during the computational sequence optimization for minimization of nucleosome binding (Curran *et al*, 2014).


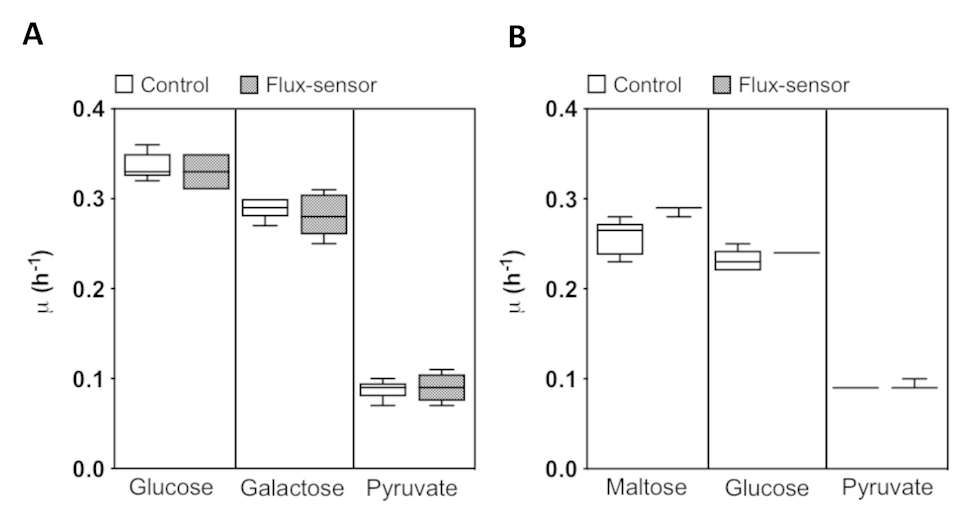


**Appendix Figure S5.** Cellular growth rate is not affected by the expression of the flux-sensor construct in WT (A) and TM6 (B) cells. Error bars represent the standard deviation of at least three replicate experiments.

**
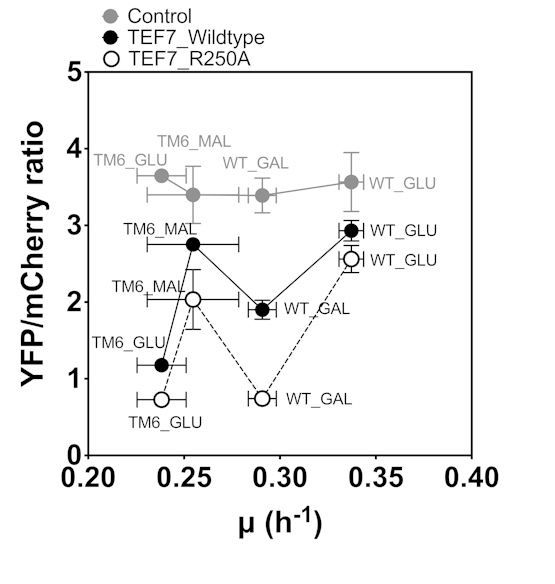
**

**Appendix Figure S6.** The flux-sensor output shows no correlation with the cellular growth rate. Error bars represent the standard deviation of at least three replicate experiments.


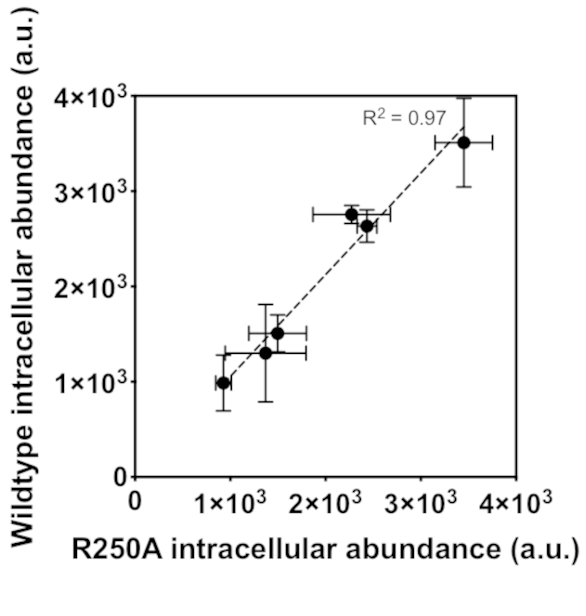


**Appendix Figure S7.** R250A intracellular levels (abundance) linearly correlate with wildtype CggR intracellular levels in WT and TM6 strains. Both CggR variants were expressed by the P_TEFmut7_. Error bars indicate the standard deviation of three independent replicates.


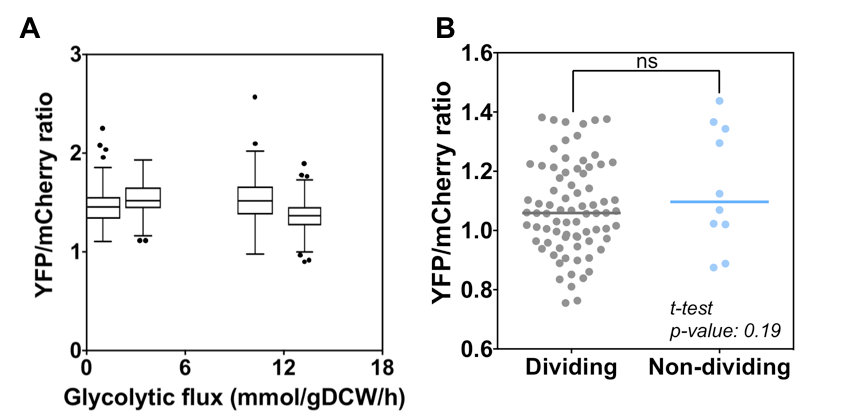


**Appendix Figure S8.** Unregulated controls of the glycolytic flux sensor. (**A**) Tukey boxplots showing the YFP/mCherry ratio of individual cells measured by microscopy for unregulated control strains that do not express the CggR repressor, cultured in different conditions. At least 35 cells were analyzed in each condition. (**B**) YFP/mCherry ratio measured by microscopy in high-flux (dividing) versus low-flux (non-dividing) TM6 unregulated control cells on 10 gL^-1^ glucose minimal medium.


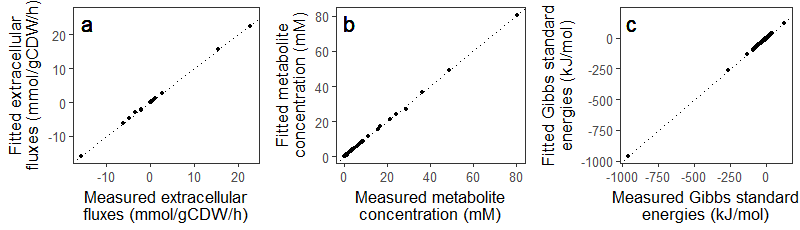


**Appendix Figure S9.** Results of the regression analysis for the six growth conditions of wildtype and TM6 strain. Fitted values from the regression analysis versus measured values; (a) extracellular rates; (b) intracellular metabolite concentrations, and (c) standard Gibbs energies of reactions.

**
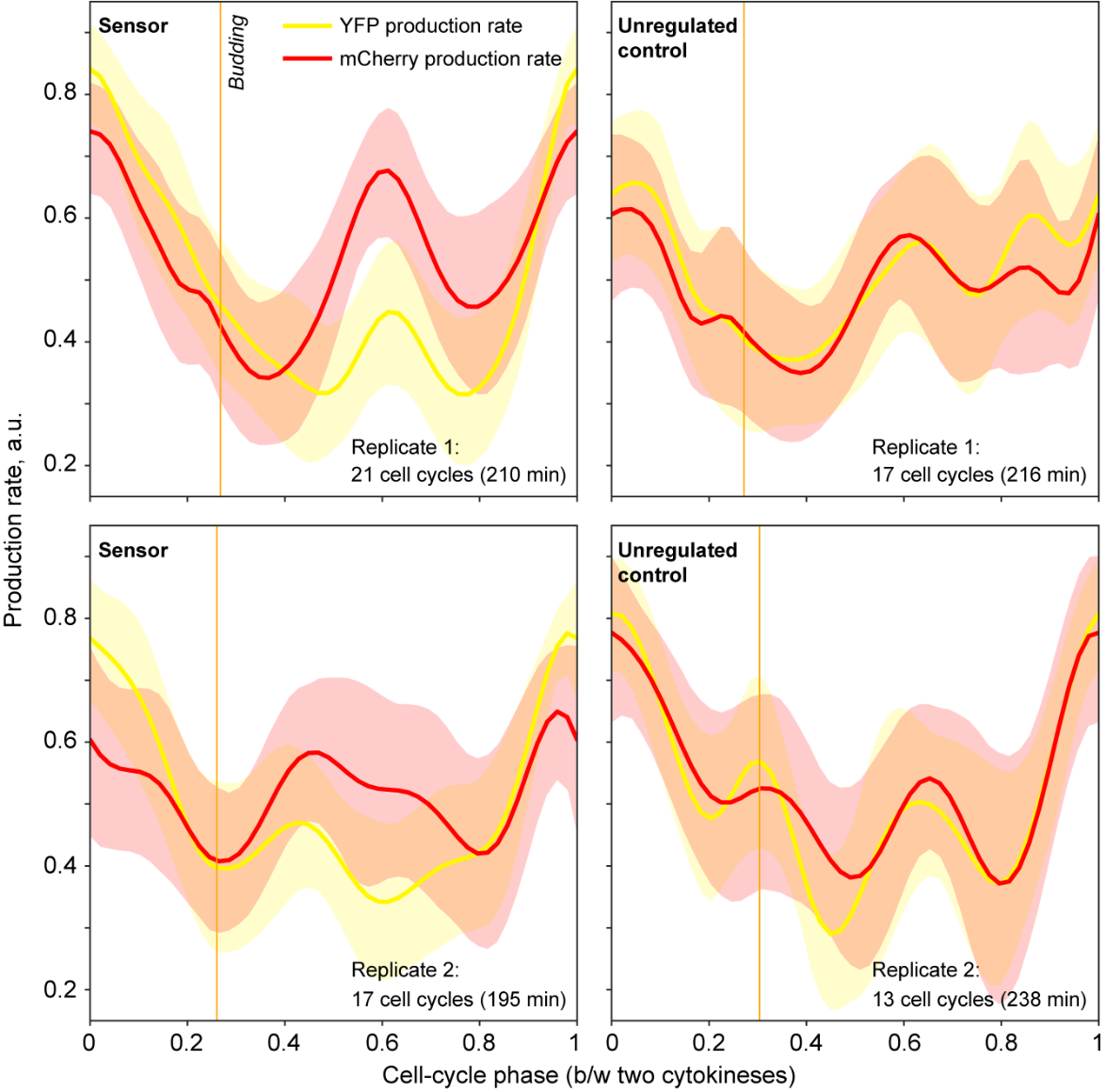
**

**B**

**A**

**Appendix Figure S10.** The production rates of YFP and mCherry are uncoupled during the cell cycle; in the biosensor-expressing strain (A), which reflects the cell-cycle dynamics of intracellular FBP concentration and glycolytic flux, but are coupled in the strain lacking CggR expression (B). The cell-cycle trajectories of the YFP and mCherry production rates were derived from YFP and mCherry fluorescence as well as cell-volume measurements, accounting for fluorescent-protein maturation. The cell-cycle tranjectories of the YFP and mCherry production rates were normalised to have the same scale (see more details in Materials and methods). Each curve represents the mean across the indicated number of cell cycles in a replicate experiment. The corresponding shaded areas denote the 95% confidence intervals of the means (bootstrapping with 5 000 iterations). The cell-cycle tranjectories of the YFP and mCherry production rates summarized here by the means were used to calculate the uncoupling summarized in Figures 7F-G. To align the cell-cycle trajectories and calculate the phase, we used the array of three cell cycle events $E=\{$cytokinesis (cyt), budding, next cyt$\}$ as reference points. Specifically, we computed the average cell-cycle-relative timing for each of these events $\bar{\varphi}^{e}$ in the following way: $\forall e\in E \bar{\varphi}^{e}=\frac{1}{N}\sum_{cc=1}^{N} \frac{t_{cc}^{e}-t_{cc}^{cyt}}{t_{cc}^{next cyt}-t_{cc}^{cyt}}$, where $N$ is the number of cell cycles in the replicate of interest, $t_{cc}^{e}$ is the time in minutes when the event $e$ happens in the cell cycle $cc$. The orange vertical lines denote $\bar{\varphi}^{budding}$ for both replicates. In the aligned cell cycles, we converted the time in minutes $t$ to the phase $\varphi_{cc}$ in the following way: $\varphi_{cc}=(\bar{\varphi}^{E\left[ i+1 \right]}-\bar{\varphi}^{E[i]})\frac{t-t_{cc}^{E[i]}}{t_{cc}^{E[i+1]}-t_{cc}^{E[i]}}+\bar{\varphi}^{E[i]}$ for $t\in\left[ t_{cc}^{E\left[ i \right]}, t_{cc}^{E\left[ i+1 \right]} \right]$ if $E\left[ i \right]=$ cyt or $t\in(t_{cc}^{E\left[ i \right]}, t_{cc}^{E\left[ i+1 \right]}]$ if $E\left[ i \right]\neq$ cyt, where $i$ is the index number of an event in the array $E$. The cell cycles used for the analysis had the duration in the interval between 150 and 300 minutes, with the mean duration presented in parentheses for each replicate experiment. The cells belonged to the TM6 strain and were cultivated on 20 gL^-1^ glucose in the microfluidic device.

**References**

Bellí G, Garí E, Piedrafita L, Aldea M, Herrero E (1998) An activator/repressor dual system allows tight tetracycline-regulated gene expression in budding yeast. Nucleic Acids Res 26: 6

Gueldener U (2002) A second set of loxP marker cassettes for Cre-mediated multiple gene knockouts in budding yeast. Nucleic Acids Res 30: e23

Hailey DW, Davis TN, Muller EGD (2002) Fluorescence resonance energy transfer using color variants of green fluorescent protein. Methods Enzymol 351: 34 – 49

Suess B, Hanson S, Berens C, Fink B, Schroeder R, Hillen W (2003) Conditional gene expression by controlling translation with tetracycline-binding aptamers. Nucleic Acids Res 31: 1853 – 1858

Voth WP (2001) Yeast vectors for integration at the HO locus. Nucleic Acids Res 29: E59 – 9
